# Changes in diffusion tensor imaging indices in basal ganglia and thalamus of patients with Relapsing-Remitting Multiple Sclerosis and relation with clinical conditions: A case-control study

**DOI:** 10.1016/j.ejro.2022.100465

**Published:** 2022-12-15

**Authors:** Mohammad Amiri, Reza Gerami, Babak Shekarchi, Amirreza Azimi, Bahador Asadi, Hamed Bagheri

**Affiliations:** a Faculty of Medicine, Aja University of Medical Science, Tehran, Iran; b Department of Radiology, Faculty of Medicine, Aja University of Medical Science, Tehran, Iran; c MS Research Center, Neuroscience Institute, Tehran University of Medical Sciences, Tehran, Iran; d Radiation Sciences Research Center, Aja University of Medical Science, Tehran, Iran

**Keywords:** MS, Multiple sclerosis, RRMS, relapsing-remitting MS, MD, Mean Diffusivities, AD, Axial Diffusivities, FA, Fractional anisotropy, RD, Radial Diffusivity, Diffusion Tensor Imaging, Multiple sclerosis, Thalamus, Basal ganglia

## Abstract

**Background:**

Multiple sclerosis (MS) is recognized as the most prevalent autoimmune abnormality of the CNS. T1WI, T2WI, and FLAIR are limited in the quantification of tissue damage and detection of tissue alterations in white and grey matter in MS. This study aimed to the evaluation of changes in DTI indices in these patients at the thalamus and basal ganglia.

**Methods:**

30 relapsing-remitting MS (RRMS) cases and 30 normal individuals were included. Conventional MRI (T2, FLAIR) was acquired to confirm NAGM in MS patients. A T1 MPRAGE protocol was used to normalize DTI images. FSL, SPM, and Explore DTI software were employed to reach Mean Diffusivities (MD), Axial Diffusivities (AD), Fractional anisotropy (FA), and Radial Diffusivity (RD) at the thalamus and the basal ganglia.

**Results:**

The FA and RD of the thalamus were decreased in healthy controls compared to MS cases (0.319 vs. 0.296 and 0.0009 vs. 0.0006, respectively) (P < 0.05). The AD value in the thalamus and the FA value in the caudate nucleus were significantly lower in MS cases than in controls (0.0009 vs. 0.0011 and 0.16 vs. 0.18, respectively) (P < 0.05). MD values in the thalamus or basal ganglia were not significantly different between groups.

**Conclusions:**

DTI measures including FA, RD, and AD have a good diagnostic performance in detecting microstructural changes in the normal-appearing thalamus in cases with RRMS while they had no significant relationship with clinical signs in terms of EDSS.

**Availability of data and material:**

Not applicable

## 1 Introduction

As an autoimmune disease, multiple sclerosis (MS) is classified as a demyelinating disease involving cerebral white matter (WM). According to estimates, there are approximately 2–3 times more women with MS than men worldwide, with a prevalence of more than 2 million cases [1] Currently, Iran is considered a high-risk area for MS [2]. Hence, research on MS diagnosis has mainly focused on the detection of MS lesions in WM in T2WI. However, recent findings have emphasized the importance of evaluating Gray Matter (GM) involvement in MS [3], [4]. MS affects the deep GM on pathological and imaging levels [5], [6]. Atrophy of the thalamus in MS is found to be related to the development of MS and disability progression [7], [8].

Conventional magnetic resonance imaging (MRI), i.e. T1w and T2w images, cannot specifically detect underlying pathologies and they cannot see the microstructural alterations in normal-appearing white and gray matter (NAWM and NAGM, respectively) in MS, more accurately [9]. The magnitude and direction of water diffusion are quantified using diffusion tensor imaging (DTI). This modality is sensitive to microstructural diffuse damage in the brain that seems to be normal on conventional MRI [10]. DTI indices including FA (fractional anisotropy), MS (mean diffusivity), RD, and AD (radial and axial diffusivities) are more specific in detecting demyelination and axonal injury than conventional MRIs [11].

A scale called the expanded disability status scale (EDSS) quantifies MS disability in terms of eight functional systems by allowing the neurologist to assign a functional system score (FSS) according to each. EDSS steps 0–3.5 refer to patients with MS who are fully ambulatory, EDSS steps 4.0–9.5 are defined by the impairment to movement. Correlation between EDSS and DTI indices of NAWM as FA and ADC were controversial[12], [13].

The current study aimed at evaluating the role of DTI indices in detecting microstructural changes of the NAGM of basal ganglia and thalamus in patients with relapsing-remitting MS (RRMS) and survey the DTI indices in relation to EDSS. It is important to know this relation because the relation of DTI indices with EDSS as a quantified clinical test had not been cleared previously as a controversial issue.

## 2 Patients and methods

### 2.1 Study population

Thirty cases(15 males and 15 females) with RRMS according to the latest revision of McDonald criteria [14] and 30(15 males and 15 females) age- and sex-matched healthy individuals were recruited To establish MS diagnosis, the McDonald criteria combine clinical and laboratory evaluations, as well as magnetic resonance imaging (MRI) data An international team led by neurosurgeon Ian McDonald published the first version of the criteria in 2001 Since then, the criteria have been updated several times, most recently in 2017. Those with claustrophobia, cerebral aneurysmal clips or pacemakers, cases with MS relapse, or who received steroid treatment 6 months before imaging were excluded. All participants signed the informed consent form.

### 2.2 Imaging

MRI was performed by GE 1.5 Tesla system (GE Optima 450 W) and a 16-channel dedicated head-coil. Patients underwent routine imaging sequences including T1, T2, FLAIR and also DTI. Protocols used for imaging included: fast spin-echo T2WI coronal: TR= 5510 ms, TE= 77 ms, FOV= 280 mm, slice thickness= 2.5 mm. FLAIR axial: TR= 8910 ms, TE= 93 ms, TI= 2489 ms, FOV= 280 mm, slice thickness= 2.5 mm. Single-Shot SE-EPI (spin echo-echo planar imaging) DTI axial: TR= 12000 ms, TE= 90 ms, slice thickness= 2 mm, FOV= 280 mm, voxel size= 2 * 2 * 2, number of signal averages 1, 30 diffusion encoding directions with 3 sets of b= 0 and b= 1000 s/mm2.

### 2.3 Image interpretation

**Fig. 1:**
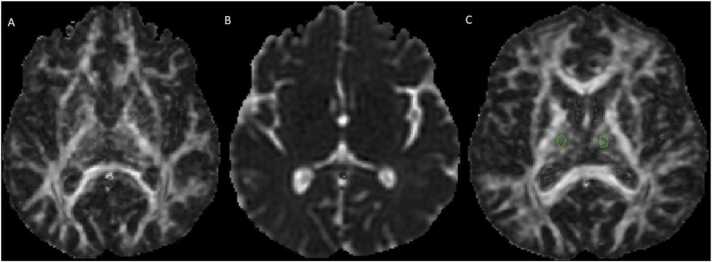
A) Fractional anisotropy image, B) Mean diffusivity image, C) ROI placement within the thalamus in fractional anisotropy image.

T2WI and FLAIR were reviewed to exclude patients with abnormal signal intensity in the deep GM (thalamus, lentiform, and caudate nuclei). Post-processing of DTI images was performed using ExploreDTI software[15]. Echo planar imaging distortion, eddy current and subject motion were then corrected using the ExploreDTI correction algorithm(Algorithm: motion correction, eddy current correction, EPI distortion correction). DTI indices were extracted from corrected data. Similar processing was also done in the control group. One researcher used B0 images to place the region-of-interest (ROI) on areas of interest. In three contiguous sections, three ROIs in each hemisphere in the thalamus, lentiform nucleus, and caudate nucleus were placed (ROI= 20 voxels in the thalamus and 10 voxels in the caudate nucleus, putamen, and globus pallidus) (Fig. 1). The widest part of the mentioned structure was considered to avoid partial volume effects. Average AD, FA, MD, and RD from three contiguous sections were considered for final analysis.

### 2.4 Data analysis

The results are described using mean±SD. Frequencies and percentages are used to describe categorical data. The data distribution was evaluated by the shapiro_wilk test. The Shapiro–Wilk test is a test of normality in frequentist statistics. Spearman’s rho test was used to assess the relationship between DTI and EDSS score. Intergroup comparisons were done using the t-test and U Mann-Whitney test. The level of statistical significance was considered to be 0.05. Data analysis was administered by SPSS V.20.

In addition, this research is confirmed by the Ethics Committee of our medical university.

## 3 Results

Thirty cases with a confirmed diagnosis of RRMS and 30 age and sex-matched normal individuals were studied. The mean age of MS cases and the control group were 37 ( ± 9.4) and 37 ( ± 8.5) years, respectively. 50% of individuals in each group were female (n = 15 in both MS patients and the control group).

Using the U Mann-Whitney test average age in two control and case groups had been compared. It was demonstrated no differences between the two groups (p = 0.807). More details as shown in Table 1.

**Table 1:** Comparison of two groups (case and control) in terms of age.

	Age	
#	patient	Control
1	49	33
2	37	47
3	38	33
4	50	43
5	47	35
6	17	20
7	49	28
8	41	28
9	36	39
10	35	38
11	33	43
12	35	34
13	49	22
14	30	33
15	24	28
16	45	40
17	28	37
18	25	42
19	48	39
20	32	34
21	50	47
22	31	38
23	24	36
24	47	50
25	37	49
26	43	18
27	30	39
28	27	50
29	47	46
30	37	43
		
Average	37.36667	37.06667
SD	9.378932	8.525553
Range	17–50	18–50
Z	-0.807	
P-value	0.807	

Table 2 represents the average FA, MD, RD, and AD in the thalamus, caudate nucleus, putamen, and globus pallidus. In control group, FA of the thalamus was significantly lower than MS cases (0.296 ± 0.013 vs. 0.319 ± 0.019, respectively) (p-value =<0.001). In contrast, average FA of caudate nucleus was decreased in MS group compared to control group (0.16 ± 0.03 vs. 0.18 ± 0.02, respectively) (p = 0.018). No significant difference was observed in MD values of thalamus and BG between MS patients and the control group (p > 0.05). RD of the thalamus was significantly increased in the MS group in comparison with the controls (0.0009 ± 0.00002 vs. 0.0006 ± 0.00002, respectively) (p-value=<0.001). AD of thalamus was decreased significantly in MS group compared to the controls (0.0009 ± 0.00003 vs 0.0011 ± 0.00003, respectively) (p-value < 0.001). The study groups were not significantly different concerning RD and AD values in BG (p > 0.05) (Table 1).

**Table 2:** Measured diffusion tensor imaging indices in MS patients and control group.

	Index	Mean ( ± SD)		P-value
		MS	Control	
Thalamus	Fractional Anisotropy	0.319 ( ± 0.019)	0.296 ( ± 0.013)	< 0.001
	Mean Diffusivity	0.0008 ( ± 3.10)	0.0008 ( ± 1.97)	0.823
	Radial Diffusivity	0.0009 ( ± 0.00002)	0.0006 ( ± 0.00002)	< 0.001
	Axial Diffusivity	0.0009 ( ± 0.00003)	0.0011 ( ± 0.00003)	< 0.001
Caudate nucleus	Fractional Anisotropy	0.16 ( ± 0.03)	0.18 ( ± 0.02)	0.018
	Mean Diffusivity	0.0008 ( ± 0.00004)	0.0007 ± (0.00005)	0.496
	Radial Diffusivity	0.0007 ( ± 0.00004)	0.0007 ( ± 0.00005)	0.283
	Axial Diffusivity	0.0010 ( ± 0.00007)	0.0008 ( ± 0.00006)	0.425
Putamen	Fractional Anisotropy	0.16 ( ± 0.02)	0.16 ( ± 0.02)	0.103
	Mean Diffusivity	0.0007 ( ± 0.00003)	0.0007 ( ± 0.00002)	0.472
	Radial Diffusivity	0.0007 ( ± 0.00003)	0.0007 ( ± 0.00003)	0.421
	Axial Diffusivity	0.0008 ( ± 0.00003)	0.0008 ( ± 0.00003)	0.648
Globus Pallidus	Fractional Anisotropy	0.35 ( ± 0.03)	0.34 ( ± 0.03)	0.348
	Mean Diffusivity	0.0009	0.0008	0.462
	Radial Diffusivity	0.0007	0.0008	0.161
	Axial Diffusivity	0.0010	0.0010	0.544

MS: Multiple Sclerosis

The mean ( ± SD) of EDSS score in patients was 2.4667 ± 0.84009 and the correlation between EDSS and DTI indices was not statistically significant. (Table 3).

**Table 3:** Relation between DTI indices and EDSS using Spearman’s rho test.

Expanded Disability Status Scale (EDSS)	Spearman’s rho			
	Index	Correlation Coefficient	P-Value	Significance
Thalamus	FA	0.089	0.638	NO
	MD	-0.212	0.261	NO
	RD	-0.155	0.413	NO
	AD	-0.029	0.879	NO
Caudate nucleus	FA	-0.203	0.282	NO
	MD	0.005	0.981	NO
	RD	-0.011	0.953	NO
	AD	0.108	0.570	NO
Putamen	FA	0.151	0.425	NO
	MD	-0.043	0.820	NO
	RD	0.006	0.973	NO
	AD	-0.033	0.861	NO
Globus Pallidus	FA	0.221	0.240	NO
	MD	-0.061	0.748	NO
	RD	-0.063	0.741	NO
	AD	0.029	0.879	NO
Age		0.329	0.076	NO

## 4 Discussion

A total of 60 individuals in two sex and age-matched case and control groups, were studied. Following conventional MRI sequences, DTI indices (i.e., FA, MD, RD, and AD) were evaluated in NAGM of the thalamus and BG. Our results showed a high value for DTI of the thalamus to detect NAGM microstructural changes in MS patients. FA, RD, and AD of the thalamus were significantly changed in the MS group in comparison to the controls. Besides, FA of caudate nuclei was significantly higher in controls than in MS cases. However, DTI of the lentiform nucleus did not show different indices between MS cases and the control group. In addition, EDSS as a quantified score had no statistical relationship to DTI indices. Due to controversy in the relationship between EDSS and DTI indices, this study was designed.

Although MRI is the most valuable modality in MS diagnosis, the association between conventional MRI findings and patients’ clinical conditions is not optimal [16]. This suggests the presence of other pathological changes in white matter or grey matter that cannot be detected in conventional MRI [17], [18]. The so-called NAGM and NAWM can be further evaluated by DTI to detect microstructural changes resulting from the proliferation of microglia, T cell infiltration, and perivascular cuffing [19], [20]. DTI is a diffusion MRI technique intended to estimate brain fiber structures by water diffusion characteristics [21]. To quantify water diffusion changes in DTI, commonly derived indices include:

a) FA which measures anisotropy of water diffusion and has a positive association with fiber density, axonal diameter, and myelination. Higher FA shows full restriction along one axis but low values are an indication of isotropic diffusion in all directions [22].

b) MD indicates the degree of diffusion, independent of direction. This is sometimes known as the apparent diffusion coefficient (ADC).

c & d) AD describes the diffusion rate along the primary axis of diffusion. Besides, RD reflects the mean diffusivity along the other two minor axes.

In a study by Homos et al. [23], the MD values of the thalamus, lentiform, and caudate nuclei were significantly higher in the MS group compared to normal individuals. Our results and Zhou et al. [24] study revealed no significant change in MD of NAGM in the MS group compared to the healthy controls. Both of these studies have generally included MS patients in every disease course; but, in this study, only cases with relapsing-remitting MS were considered eligible. Woitek et al. study revealed higher MD values in the putamen in primary progressive MS compared to secondary progressive MS patients [25]. Hence, specifying the disease course is important in the interpretation of DTI measures in MS patients and the discrepancy between our results and those of Homos et al. can be attributed to the difference in inclusion criteria.

Thalamus is an information relay center and its role in MS development is correlated with various clinical findings such as fatigue and cognitive impairment [26]. Our results revealed a high value for DTI of the thalamus in relapsing-remitting MS. FA and RD of the thalamus were significantly higher in MS patients and AD was significantly higher in healthy cases. Generally, the high density of the ordered structures (axonal fibers) is associated with high FA; thus, a lower FA is expected in damaged tissues [22]. However, FA changes in the thalamus in MS are controversial. Some studies have reported decreased FA in the thalamus of MS patients [27], [28] while others showed increased FA values [3], [23], [29], [30].

Our results also showed significantly decreased FA value in the caudate nucleus of MS patients while Hasan et al. revealed an approximately 9% larger FA value in the RRMS group relative to controls [31]. The present controversy in the change of FA values in NAGM of MS patients can be attributed to the degree of pathological changes of NAGM at the time of imaging. Microglial activation results in dendrites loos and bipolar orientation which causes an increased FA [32] but inflammatory processes result in decreased FA [33]. Depending on the dominancy of each of mentioned processes, the FA values may increase or decrease in MS patients. These findings emphasize the importance of the change in FA values in the evaluation of microstructural changes in MS.

Our results showed significantly increased and decreased RD and AD values in MS patients' thalamus. Increased thalamus AD and RD values have been reported in previous studies [27], [34]. Underlying tissue structure contributes to the precise interpretation of the AD and RD alternations [35]. Moreover, the GM inflammation level at imaging time and also heterogenicity of sample size and ROI selection may attribute to decreased AD in our study. Regardless of the direction of alteration of AD and RD, these results suggest the role of measuring these indices in the evaluation of thalamus microstructural injuries in MS patients.

The relation between Expanded Disability Status Scale (EDSS) and DTI indices is in an aura of ambiguity. Some studies revealed a significant relationship but others did not. Fink et al. in 2010 surveyed DTI changes in PRMS in two cases (53 patients) and control (15 healthy individuals) groups. The median EDSS was 2.5 while none of the DTI indices was related to EDSS. [36] In another study Griffin et al. examined DTI changes in relation to EDSS in 28 patients and 27 healthy people and demonstrated no significant relationship between EDSS and DTI indices. [37]These two studies were similar to ours. In contrast, some studies revealed a significant relationship. Benedetti et al. surveyed 40 benign MS, 28 secondary progressive MS, and 18 healthy individuals and demonstrated that EDSS had a significant relationship with average cord FA. [38] Another study showed similar results. [39], [40], [41], [42], [43] EDSS is an index that is generally influenced by motor dysfunctions so in the early stages maybe not be altered. Also, a clinical correlation between diffusion changes and disability is less likely to be found when diffusion changes are evaluated in the whole brain [44]. Maybe more studies are needed to evaluate these differences.

Our study suffers from some limitations, including the lack of longitudinal studies to better understand changes in DTI measures of NAGM. Also, due to limitations regarding the study’s scope, clinical correlation with DTI measures was not studied. Moreover, this study used a 1.5 Tesla MRI machine which can affect the accuracy of DTI measures, and future studies with 3 T MRI are recommended. In conclusion, thalamus DTI has a good diagnostic performance in relapsing-remitting MS patients. Based on our results and those of previous studies, various patterns of AD, RD, MD, and FA alteration may be seen in the thalamus DTI of these patients but a normal DTI measure is less likely. Future studies should focus on different clinical courses of MS and also correlate DTI measures with the clinical conditions of the patients.

## Funding

This research did not receive any specific grant from funding agencies in the public, commercial, or not-for-profit sectors.

## Ethics approval and consent to participate

Ethics committee of AJA University of Medical Sciences approved this descriptive cross-sectional study.

## CRediT authorship contribution statement

Dr R G supervised MRI study on all patients and recorded data related to MRI information. Dr M A and B S and AA extracted all non-imaging information from patients by interview. B A and Dr H B analyzed interpreted the patient data. All authors read and approved the final manuscript.

## Competing interests

The authors declare that they have no competing interests.
